# Delineating and identifying long-term changes in the whooping crane (*Grus americana*) migration corridor

**DOI:** 10.1371/journal.pone.0192737

**Published:** 2018-02-15

**Authors:** Aaron T. Pearse, Matt Rabbe, Lara M. Juliusson, Mark T. Bidwell, Lea Craig-Moore, David A. Brandt, Wade Harrell

**Affiliations:** 1 Northern Prairie Wildlife Research Center, U.S. Geological Survey, Jamestown, North Dakota, United States of America; 2 Nebraska Ecological Services Field Office, U.S. Fish and Wildlife Service, Wood River, Nebraska, United States of America; 3 U.S. Fish and Wildlife Service, Lakewood, Colorado, United States of America; 4 Canadian Wildlife Service, Environment and Climate Change Canada, Saskatoon, Saskatchewan, Canada; 5 U.S. Fish and Wildlife Service, Austwell, Texas, United States of America; Sichuan University, CHINA

## Abstract

Defining and identifying changes to seasonal ranges of migratory species is required for effective conservation. Historic sightings of migrating whooping cranes (*Grus americana*) have served as sole source of information to define a migration corridor in the Great Plains of North America (i.e., Canadian Prairies and United States Great Plains) for this endangered species. We updated this effort using past opportunistic sightings from 1942–2016 (*n* = 5,055) and more recent (2010–2016) location data from 58 telemetered birds (*n* = 4,423) to delineate migration corridors that included 50%, 75%, and 95% core areas. All migration corridors were well defined and relatively compact, with the 95% core corridor averaging 294 km wide, although it varied approximately ±40% in width from 170 km in central Texas to 407 km at the international border of the United States and Canada. Based on historic sightings and telemetry locations, we detected easterly movements in locations over time, primarily due to locations west of the median shifting east. This shift occurred from northern Oklahoma to central Saskatchewan at an average rate of 1.2 km/year (0.3–2.8 km/year). Associated with this directional shift was a decrease in distance of locations from the median in the same region averaging -0.7 km/year (-0.3–-1.3 km/year), suggesting a modest narrowing of the migration corridor. Changes in the corridor over the past 8 decades suggest that agencies and organizations interested in recovery of this species may need to modify where conservation and recovery actions occur. Whooping cranes showed apparent plasticity in their migratory behavior, which likely has been necessary for persistence of a wetland-dependent species migrating through the drought-prone Great Plains. Behavioral flexibility will be useful for whooping cranes to continue recovery in a future of uncertain climate and land use changes throughout their annual range.

## Introduction

Delineating species’ ranges is a common and long-standing practice in ecology. Techniques and data vary in sophistication from basic range maps generated from expert opinion and opportunistic sightings to species distribution models derived from intensive field data and remotely sensed data [1]. Knowledge of where a species resides provides a wealth of information to make better conservation and management decisions [2]. Conservation planning activities generally require knowing where to protect habitats needed for sustainable populations. Range and distribution also allow evaluation of impacts and responses to climate and land use changes by species as well as identification and determination of exposure to potential threats [3].

Species and populations have distinct spatial distributions and ranges based on biotic and abiotic factors. Changes in conditions over part or all of their range can result in distribution shifts. Such shifts can manifest as an increase or decrease in area, or include different geographic space with no net change in range size. Many species have experienced range contractions due to habitat loss and conversion of land to uses and habitat types that no longer support them (e.g., effects of conversion of grasslands to croplands on grassland birds; [4]). Range expansions also occur in response to habitat change; mallards (*Anas platyrhynchos*) in North America have increased in numbers along the Atlantic Coast in conjunction with changes in land use [5]. Along with land use change, climate change has been identified as causing range shifts [6]. Shifts towards the poles and to higher elevations have been common documented trends [7] and, for migratory birds, research efforts have focused primarily on shifts in breeding and wintering areas [6,8,9]. In contrast, changes to areas used during migration have received less attention, even though these areas are essential to migratory birds. Detecting and tracking changes in spatial distributions will assist managers in conserving species and habitats in these new places.

The whooping crane (*Grus americana*) is a listed endangered species, protected under federal legislation in Canada and the United States. With their historic range encompassing the Great Plains of North America (i.e., Canadian Prairies and United States Great Plains) and beyond, the species experienced an extreme range reduction along with near extinction [10]. Past and current recovery actions have relied upon knowledge of where remaining individuals exist throughout the year. Location of wintering areas was the basis for establishment of Aransas National Wildlife Refuge in Texas [10,11]. Breeding grounds at Wood Buffalo National Park were unknown until 1954 and have been defined and monitored since. Migration areas between breeding and wintering areas have been identified primarily from opportunistic sightings in Canada and the United States, and a radio telemetry study conducted in the late 1970s and early 1980s confirmed these earlier findings [12]. Monitoring movements during migration and identifying and protecting essential habitat have been identified as a recovery action for whooping cranes [13].

Although location data for migrating whooping cranes has been collected for decades, few efforts to delineate a migration corridor between breeding and wintering areas have been completed. A previously developed fixed-width corridor was primarily designed to determine threats of power lines to whooping cranes in the United States and Canada [14], which was subsequently updated in the United States part of the corridor with a similar analytical approach [15]. These efforts have provided a basic corridor location, but results from a past radio telemetry study suggest variation in width of the corridor, as available habitats and presence of traditional staging areas vary with latitude [12]. An effort to mark and track whooping cranes using satellite telemetry began in 2009, with majority of data collected 2010–2016 [16]. Given newly available data, there is opportunity to develop a contemporary migration corridor to further support recovery efforts of this iconic endangered species. Our objectives were to: 1) delineate a current migration corridor that varies geographically in size and shape and provides estimates of uncertainty, as any delineation is an estimate and requires some acknowledgment of uncertainty, using available whooping crane locations during migration; and 2) assess changes to the migration corridor that may have occurred over the past 8 decades.

## Methods

### Ethics statement

Capture and marking were conducted under Federal Fish and Wildlife Permit TE048806, Texas research permit SPR-1112-1042, Aransas National Wildlife Refuge special use permit, Canadian Wildlife Service Scientific Permit NWT-SCI-10-04, Parks Canada Agency Research and Collection Permit WB-2010-4998, and Northwest Territories Wildlife Research Permits WL004807, WL004821, and WL500051. Procedures were approved by Animal Care and Use Committee at Northern Prairie Wildlife Research Center and Environment Canada’s Animal Care Committee.

### Species and study area

Whooping cranes of the Aransas-Wood Buffalo population represent the lone remnant population of this endangered species [13]. Nesting habitat destruction and shooting were the primary causes of historic population decline [10]. Whooping cranes breed, winter, and migrate across a variety of habitats, including inland wetlands, coastal marshes, lakes, rivers, grasslands, and agricultural fields. They breed along borders of the Northwest Territories and Alberta, Canada, in and around Wood Buffalo National Park, and winter primarily along the Texas Gulf Coast near Rockport, Texas, USA [10]. Twice annually, cranes migrate between these sites, traversing approximately 4,000 km [12]. The center of their migration corridor passes through Canadian provinces of Alberta and Saskatchewan and the USA states of North Dakota, South Dakota, Nebraska, Kansas, Oklahoma, and Texas [15,16]. This region is within the Great Plains and classified primarily a grassland biome. Mixed-grass prairie historically dominated, with a precipitation gradient from a more arid west to a more mesic east [17].

The majority of land in the Great Plains is in private ownership and used primarily for agricultural production, including annual crops grown for food, livestock feed, and biofuels and pasture and haylands for ranching operations [17]. This region also includes landscapes with high abundance of wetland features, such as Prairie Pothole Region, Nebraska Sandhills, Rainwater Basin Area, and Playa Lakes Region [18]. In addition, numerous river drainages are situated across the Great Plains, including Missouri River, Platte River, Arkansas River, Red River, and Saskatchewan River systems. These wetlands and rivers support a diverse array of aquatic plant and animal communities and support millions of migratory waterfowl and waterbirds. Whooping cranes use wetlands and rivers as roosting and foraging sites during migration [19,20].

### Field methods

The Canadian Wildlife Service (CWS) and U.S. Fish and Wildlife Service (USFWS) have individually maintained databases of opportunistic whooping crane sightings made throughout the Great Plains [15,21]. Members of the public, professional biologists, and conservation officers have reported opportunistic sightings since the 1960s in Canada and 1975 in the United States, although some data predate organized programs. In Canada, a telephone system was set up in 1986 to receive reports and has served as primary source of information since its inception. The CWS and USFWS have classified opportunistic sightings as confirmed, probable, or unconfirmed, and verified reports by interviewing observers, documenting by photographs, and confirming sightings by qualified staff. In delineating the migration corridor, we used only opportunistic sightings that have been classified as confirmed. We also excluded sightings in Canada that occurred during summer, which we defined as dates between 25 May and 20 August, based on migration timing from telemetry data. We removed these locations because we were interested in identifying a corridor where individuals were actively migrating. Observations between 25 May and 20 August likely occurred during sedentary summering periods and birds may use different locations and habitats at these times compared to during migration. Sightings represent crane groups (i.e., 1 or more individuals). Opportunistic sightings have been collected with variable levels of spatial accuracy, and many have been recorded based on landmarks or using Public Land Survey System (i.e., range, township, section; [15]). Sightings in the United States have been archived in degrees, minutes, and seconds or decimal degrees (0.000001) of latitude and longitude, using North American Datum of 1983. Sightings in Canada have been archived in decimal degrees (0.000000001) of latitude and longitude, using North American Datum of 1983.

Between 2009 and 2014, we captured 68 cranes (~20% of Aransas–Wood Buffalo population) and attached platform transmitting terminals with global position system (GPS) capabilities (North Star Science and Technology LLC, Baltimore, Maryland, USA and Geotrak, Inc., Apex, North Carolina, USA) at and near Wood Buffalo National Park and sites along Texas Gulf Coast. Capture and marking details were described in Pearse et al. [16]. Transmitters recorded 4–5 GPS locations daily at equal time intervals, which provided daytime and nighttime locations. We initially inspected GPS locations for errors occurring during collection or transmission on the Argos satellite system [22]. We performed multiple assessments to determine plausibility of locations and omitted locations outside expected time sequences, with an implausible rate of displacement (>100 km/h), or forming an acute angle (<5°) at distances greater than 50 km (distance/angle; [23]). We recognized locations collected during migration (spring and autumn) based on manual inspection of movement patterns with respect to time of year [16,24]. Only 58 of 68 marked cranes provided locations during migration. We classified migration locations as occurring in flight when instantaneous velocity reading was greater than 2.6 m/s. Ground locations were organized into individual stopover sites for each whooping crane by identifying clusters of locations based on distance, movement pattern, and manual inspection. In general, we delineated stopover sites if birds moved >15 km between ground-based locations. After identifying a set of locations as a unique stopover location, we calculated its centroid for use in analyses along with all locations collected while birds were in flight between centroids. Locations were originally collected in decimal degrees of latitude and longitude (0.0001) using World Geodetic System 1984 datum.

### Data analyses

Before beginning analyses, we projected opportunistic sightings and telemetry locations to the North America Equidistant Conic projection and used North American Datum of 1983. Also, we divided datasets into equally-sized analysis windows along the y-axis (i.e., north-south). We tested multiple window heights from 50 to 700 km at 50 km increments. Our goal was to find an analysis window that produced a migration corridor providing a good fit to the data with the least complex shape (i.e., concurrently maximize data fit and smoothing) in order to avoid overfitting corridor models. For this fitting process, we estimated a 95% core area (methods described below) and used 1,000 bootstrap estimates to determine which resulting migration corridors using window heights from 50 to 700 km contained 95% of data, meeting our criterion for data fit. We determined shape complexity of migration corridors using fractal dimension index [25]. This index ranges from values of 1–2, and we were interested in minimizing this value, as lower values represent less complex shapes.

Initially, we defined separate migration corridors using opportunistic sightings from 1942–2016 and telemetry data from 2010–2016 (S1 Fig). Within analysis windows of size determined based on above criteria, we calculated percentiles of the coordinates’ easting (i.e., x dimension) that corresponded to the 50% core (i.e., area between 25th and 75th percentiles), 75% core (i.e., area between 12.5th and 87.5th percentiles), and 95% core (i.e., area between 2.5th and 97.5th percentiles). Federal agencies tasked with managing whooping cranes identified these core areas and corresponding percentiles as most useful for current and future conservation and recovery efforts as well as consistency with past corridor designations [15]. We combined percentiles derived from separate opportunistic and telemetry datasets by calculating averages, weighted by dataset-specific sample sizes from each analysis window. We connected final percentile vertices together to form lines outlining western and eastern extents of corridors. We converted these lines to a polygon that represented 50, 75, and 95% core migration corridors. After estimating point estimate percentiles within each analysis window, we generated 95% confidence limits by bootstrapping opportunistic and telemetry datasets 1,000 times, averaging point estimate percentiles, and extracting 2.5th and 97.5th percentiles as lower and upper confidence limits for each associated point estimate. We created polygons based on these confidence limit values as described above to define areas of uncertainty.

We explored long-term changes in the migration corridor location and characteristics by conducting analyses along the corridor using opportunistic sightings and telemetry locations and the same window width as in developing migration corridor polygons. Because of potential bias in opportunistic sightings [15,26], we initially compared medians derived from each dataset in overlapping years (2010–2016) at each analysis window. We estimated 95% confidence limits for these median differences by extracting 2.5th and 97.5th percentiles of 1,000 bootstrapped estimates at each analysis window.

Using a Bayesian framework we explored temporal changes in characteristics of the whooping crane corridor. Within this framework, we considered opportunistic sightings as prior information, as they had been collected over numerous decades, but may have inherent biases [26]. We used this ‘prior’ information and the likelihood of the telemetry data to estimate posterior probability distributions, which we used as inference. Initially, we used a general linear model to explain variation in east-west position of opportunistic sightings over time (Proc GENMOD, SAS Institute, Inc., Carey, North Carolina, USA). The x-coordinate of each sighting served as response variable, year of sighting as a continuous explanatory variable (year 0 = 1942), and analysis window as a categorical variable. We evoked a Bayesian analysis with the BAYES statement, which used Markov chain Monte Carlo methods with Gibbs sampling (2,000 burn-in and 10,000 Markov chain samples). We used an uninformative uniform prior to analyze opportunistic sightings. We calculated means and variances from posterior probability distributions of each parameter, and used these means and variances to define parameter priors as normal distributions in a subsequent and final analysis using telemetry locations. We used 95% credible intervals from posterior distributions of slope parameters for year of sighting (i.e., rates of change) to determine if values were different from 0 due to chance alone. We also investigated temporal changes in migration corridor width by determining median corridor location based on opportunistic and telemetry data and then calculating distance of each location from this centerline to points overall and distance west or east of the line. We used the Bayesian framework described above to test for changes in the absolute value of distance from the centerline overall and separately for locations west and east of centerline. For display, we calculated point estimates of model predictions for years 1980 and 2014, which represented 10^th^ and 90th percentiles of sightings by observation year.

**Fig 1:**
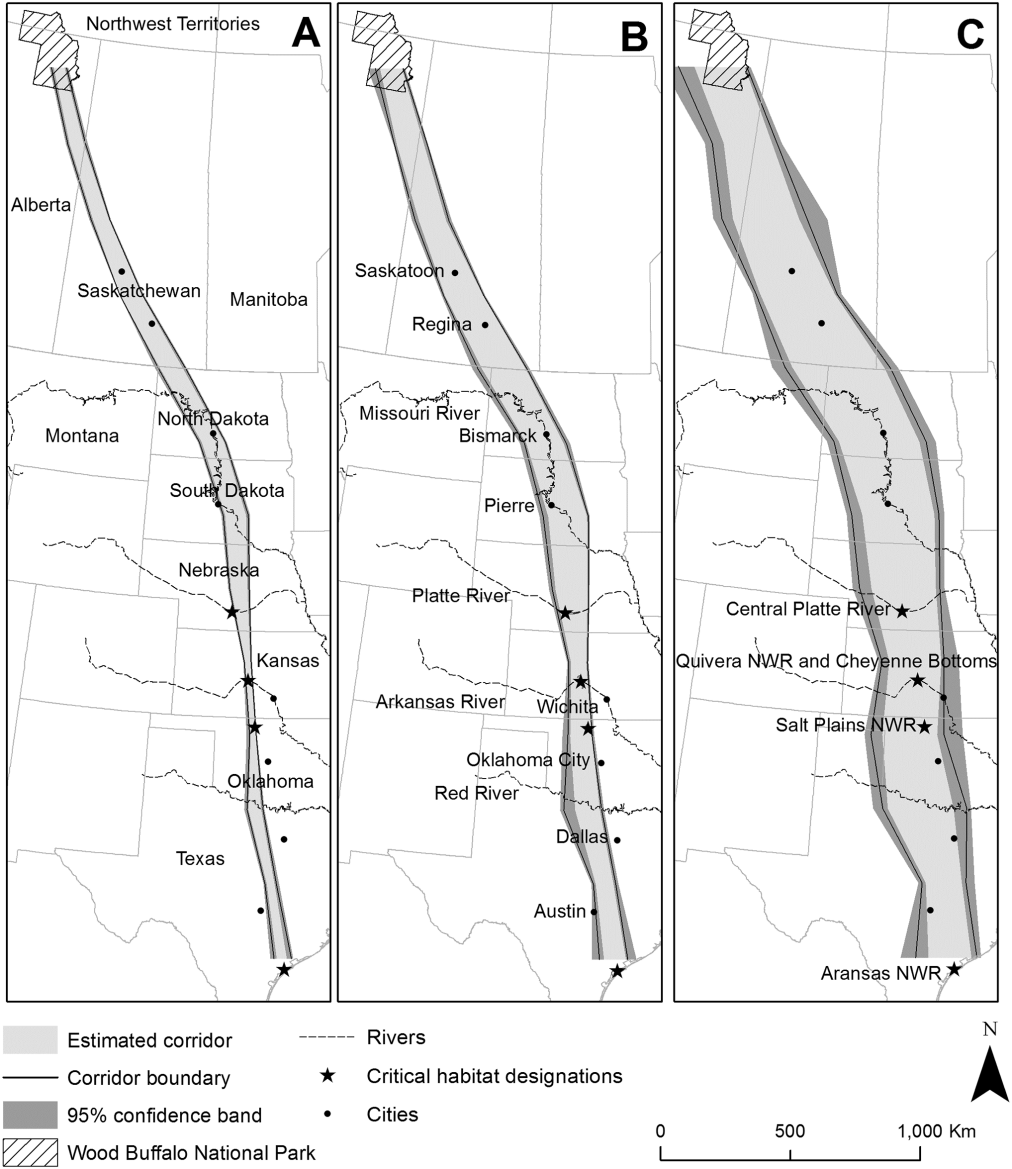
Migration corridors for whooping cranes of the Aransas-Wood Buffalo population, delineating 50% core (A), 75% core (B), and 95% core migration areas, with 95% confidence bands.

Unless otherwise noted, we calculated average widths of corridors, confidence bands, and other summaries using the 13 analysis windows as sample points. We used ArcGIS 10.5.1 (ESRI, Inc., Redlands, California, USA) for geospatial analyses and SAS 9.4 (SAS Institute, Inc., Carey, North Carolina, USA) for all other summarizations and calculations. Data used in this analysis and geospatial data of whooping crane migration corridors depicted in Fig 1 are available in the public domain from the USGS ScienceBase data repository [27,28].

## Results

We compiled 9,478 whooping crane locations from opportunistic sightings (53%) maintained by CWS and USFWS and GPS locations from 58 telemetered cranes (47%). Locations and opportunistic sightings were more common during autumn (57%) compared with spring migration (43%). We used sightings gathered in Canada between 1943 and 2016 and sightings in the United States between 1942 and 2016. Telemetry locations were gathered between 2010 and 2016. Median year of all data was 2011 and median year of opportunistic sightings was 2001.

Migration corridors created using analysis windows 50–300 km in height contained 95% of data. Fractal dimension indices for corridors using 50–300 km window sizes ranged from 1.048–1.081. The corridor created using a 300-km window had the lowest index value and was selected for use in subsequent analyses.

Migration corridors derived from opportunistic data only were generally wider than those developed solely from telemetry data (S1 Fig). The combined 95% core migration corridor of Aransas-Wood Buffalo whooping cranes bisected states of Texas, Oklahoma, Kansas, Nebraska, and South Dakota, tracking approximately north. At the North and South Dakota border, the corridor began tracking northwest through North Dakota, the northeastern corner of Montana and southwestern corner of Manitoba, Saskatchewan, and the northeastern portion of Alberta before ending at Wood Buffalo National Park (Fig 1). By province or state, the largest area of 95% corridor occurred in Saskatchewan (25.0 million ha) and Texas (15.3 million ha). The 95% corridor encompassed 64% of land area of North Dakota and 38–61% of areas of Saskatchewan, South Dakota, Nebraska, Kansas, and Oklahoma. The 95% core migration corridor intersected areas with high wetland densities including portions of Prairie Pothole Region, Nebraska Sandhills, Rainwater Basin Area, and Playa Lakes Region.

We represented the 50% core migration corridor with a 24.8 million ha polygon that intersected all areas designated as critical habitat for whooping cranes in the United States (Fig 1). This corridor had an average width of 68 km (SD = 26). It was narrowest in northern Oklahoma (25 km) and widest at the United States and Canada border (105 km). The 75% core migration corridor was represented by a 47.6 million ha polygon with an average width of 130 km (SD = 35). This corridor was narrowest in central Kansas (73 km) and widest near the international border (190 km). The 95% core migration corridor was represented by 107.1 million ha area polygon with an average width of 294 km (SD = 62). The corridor was narrowest in central Texas (170 km) and widest at the international border (407 km).

When estimating uncertainty in corridor boundaries, widths of confidence bands varied by location and corridor size (Fig 1). The 50% core migration corridor had confidence bands with an average width of 13 km, with slightly narrower bands in the eastern (13 km) compared with western edge (14 km). The 75% core migration corridor had estimated confidence bands that averaged 23 km. The eastern side of the corridor had narrower bands on average compared with the western side (eastern = 17 km; western = 29 km). The estimated confidence bands of the 95% core migration corridor were largest, averaging 60 km. As with other corridors, the eastern edge had narrower band widths on average than the western side (54 vs. 66 km average width).

**Fig 2:**
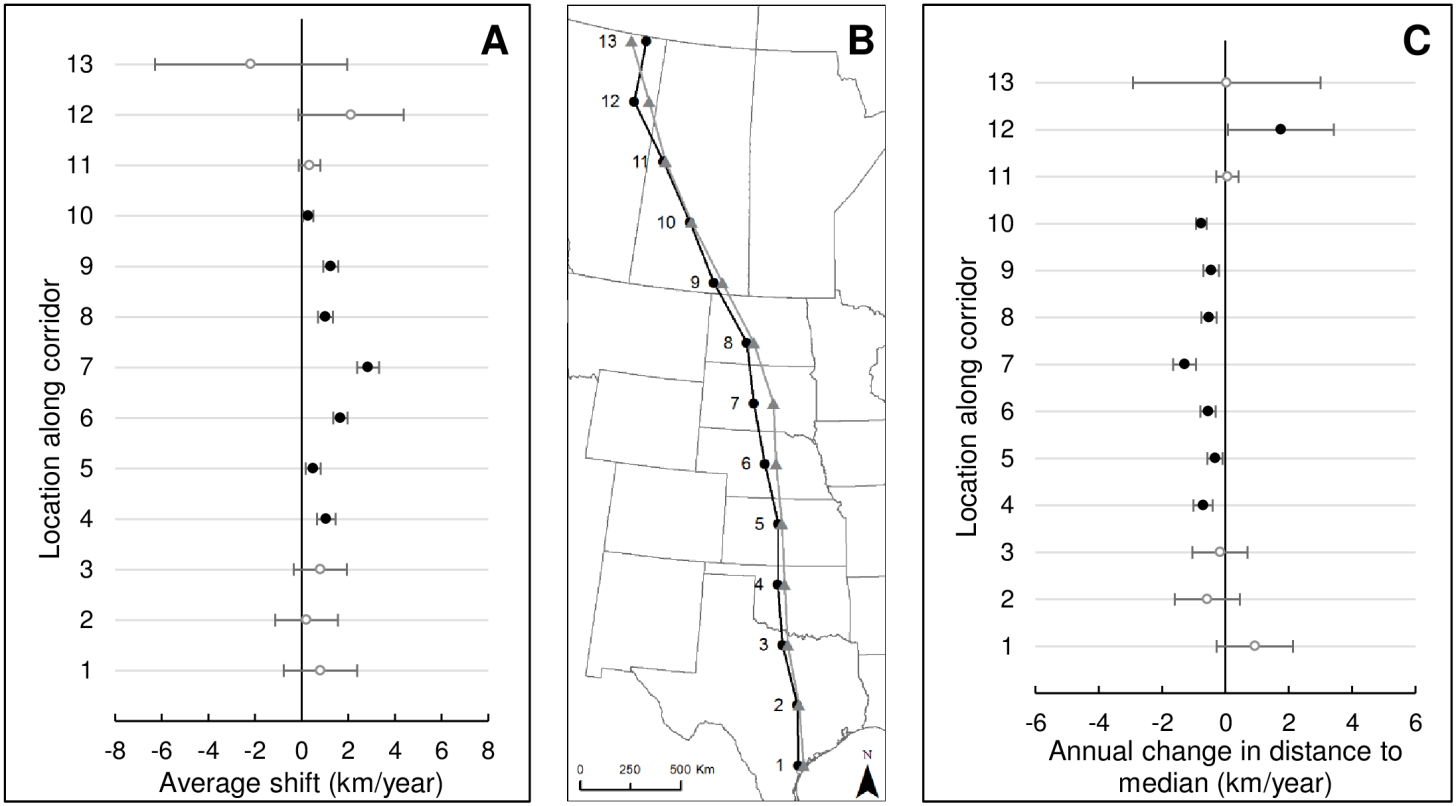
Temporal change in center and width of the whooping crane migration corridor based on opportunistic sightings and telemetry locations, 1942–2016. Estimated average east-west shift in 13 300-km analysis windows along the migration corridor (A, km/year). Open circles represent estimates where the 95% credible intervals included 0 and closed symbols represent estimates where the 95% credible intervals do not include 0. Positive values reflect eastward change, whereas negative values reflect westward movement. Predicted average locations (B) along the migration corridor during 1980 (black circles and line) and 2014 (gray triangles and line). Numbers at each point identify centers of 13 300-km windows used in analyses. Annual average change and 95% credible intervals in distance of locations relative to median line of the whooping crane migration corridor (C).

We compared medians calculated from opportunistic sightings and telemetry locations from 2010 to 2016 for 7 analysis windows in which there were ≥30 opportunistic sightings (analysis windows 4–10; Fig 2). Medians from opportunistic sightings during 2010–2016 were consistently east of telemetry locations (0.6–15.1 km). Bootstrap estimated 95% confidence limits around these estimates included 0 for all windows, expect for window 6, where the median difference was 9.6 km (95% confidence limits = 1.8–21.4 km).

In 12 of 13 300-km analysis windows, whooping crane locations shifted eastward with time, and for 5 analysis windows, 95% credible intervals did not include 0 (Fig 2A). The greatest magnitude shift occurred in central South Dakota (2.8 km/year; 95% credible interval = 2.4–3.3). We observed consistent easterly shifts of whooping crane sightings from northern Oklahoma to central Saskatchewan, at an average rate of 1.2 km/year. Between 1980 and 2014, an eastward shift among sightings averaged 28 km (maximum = 97 km in central South Dakota; Fig 2B). Between central Oklahoma and central Saskatchewan, the easterly shift averaged 42 km (9–97 km).

**Fig 3:**
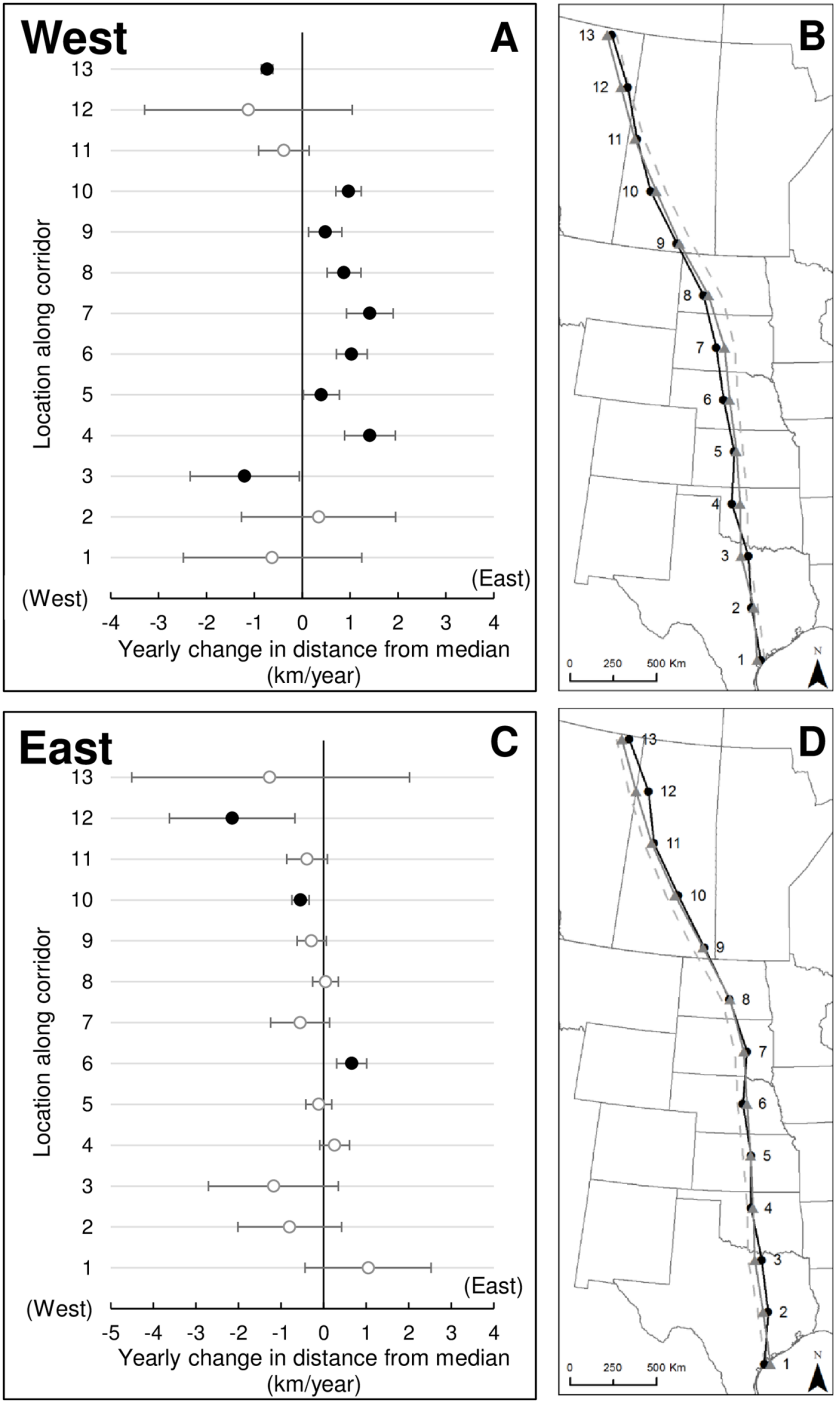
Temporal rate of change in location (± 95% credible intervals) from median line of the whooping crane migration corridor based on opportunistic sightings and telemetry locations, 1942–2016. Estimated rate of shift at 13 analysis windows for locations that were west (A) and east (C) of the median line of the migration corridor. Open circles represent estimates where 95% credible intervals included 0 and closed symbols represent estimates where 95% credible intervals do not include 0. Predicted distance of locations along the migration corridor during 1980 (black circles and solid line) and 2014 (gray triangles and line) west (B) and east (D) of the corridor center. The gray dashed line represents the migration corridor median line and numbers at each point identify centers of 13 300-km windows used in analyses.

When comparing location distances to their median, temporal shifts toward the median were more consistent than those outward, indicating a narrowing over most of the migration path (Fig 2C). Seven analysis windows had movements towards the median, wherein the 95% credible interval of the slope did not include 0, with an average -0.7 km/year rate of change and maximum -1.2 km/year rate of change in central South Dakota. One analysis window indicated a temporal shift away from the median (i.e., widening corridor) in Alberta at a rate of 1.8 km/year. For locations west of the median, we found 8 analysis windows with easterly shifts and, for 7 windows, the decrease was significantly different from 0 (Fig 3A). The greatest magnitude shift was in central South Dakota (1.4 km/year; 95% credible interval = 0.9–1.9). Between northern Oklahoma and central Saskatchewan, we observed consistent rates of shifting closer to the median (i.e., eastward) at an average rate of 0.9 km/year. In 2 analysis windows in northern Alberta and northern Texas, locations shifted outward from the median. For locations east of the historic median, we observed significant changes in 3 analysis windows, 2 decreasing (westerly) and 1 increasing (easterly). Westerly shifts occurred in central Saskatchewan and northern Alberta, whereas an easterly shift occurred in central Nebraska (Fig 3C and 3D). The remainder showed changes close to 0 or were not estimated with enough precision to be distinguished from 0.

## Discussion

We delineated a well-defined and relatively narrow migration corridor for the Aransas-Wood Buffalo population of whooping cranes, the only self-sustaining population of this endangered species. This migration corridor generally included areas of the Great Plains similar to those identified by past work [12,14,15]. Our corridor provides a robust estimate that incorporated opportunistic sightings that were used in past corridor estimates and GPS locations from more current marking efforts. The most restricted migration corridor, depicting 50% core area, intersected places designated as critical habitat within the United States [13], providing continued assurance that these areas identify locations where whooping cranes have continued to use and pass over during their semi-annual journeys. Defining where whooping cranes are most likely to occur during migration can be useful for managers to target conservation, mitigation, and recovery actions and assess threats. Just as sufficient resources are required at wintering and nesting areas to promote population growth, stopover sites with suitable roosting sites and food promote survival during migration and reproduction once reaching breeding areas. Grassland and wetland losses in the Great Plains have been extensive and, because of limited financial resources, efforts to protect and restore these ecosystems must be strategic [29]. Location within the migration corridor could serve as evidence of high potential use by whooping cranes and be used to prioritize easement purchases or restoration efforts [2], or to identify additional critical habitat under federal legislation. For example, predicted densities of upland-nesting waterfowl have factored into where wetland easements and conservation reserve program efforts have been placed within U.S. Prairie Pothole Region [30–32]. Agencies also can use the corridor to assess potential impacts of proposed projects when they are tasked with determining potential short and long-term adverse effects to whooping cranes. For example, certain threats like those posed by infrastructure related to wind energy production may have high levels of overlap and have potential to cause negative impacts (e.g., [33]) or may have more limited co-occurrence and potential for interactions (e.g., [34]). Understanding the spatial distribution of threats in relationship to the migratory corridor is important to mitigating those threats. Predictions of where those threats may arise in the future should be used to better implement conservation strategies for migrating whooping cranes.

A previous effort found that approximately 95% of sightings in Canada and the U.S. were included within a 320-km wide corridor [14]. Our 95% core migration corridor had a similar average width of 294 km, although our corridor varied in width by ±40%, providing greater accuracy by including areas where cranes spread out longitudinally and excluding areas where they used a narrower pathway. We suspect latitudinal differences were due to differences in wetland density and distribution throughout the migration corridor. Areas with lower densities of wetlands, specifically in the southern and central Great Plains, likely provide fewer stopover options for whooping cranes [35]. Moreover, reservoirs and stock ponds were conspicuous in these regions, potentially providing more permanent surface water resources [36]. These spatial patterns and wetland dynamics would require and allow for a more constrained corridor in areas used by many birds, especially in Oklahoma and Kansas. In contrast, density, distribution, and ephemeral nature of wetlands occur north of the Platte River in Nebraska and continue into the Nebraska Sandhills and Prairie Pothole Region in South Dakota, North Dakota, and Saskatchewan. A wider corridor in these areas may be a result of cranes searching for suitable wetlands across years of differing wetland conditions.

Numerous migratory bird species have responded to environmental changes by adapting when and where they migrate [37]. Many of these modifications were phenology changes, where birds initiated migrations and arrived on breeding areas earlier than in the past [38–41]. Changes to spatial distribution during migration also have occurred, where birds occupied different or novel areas [42]. Decades of whooping crane sightings have revealed detectable changes in their migration route across the Great Plains and these changes have been supported by more recent telemetry data (S1 Table). An easterly centerline shift, most pronounced in South Dakota but apparent from northern Oklahoma to Saskatchewan, arose primarily because locations west of the long term median line shifted eastward over the past few decades. Conversely, we did not detect a strong trend in locations east of the median line moving eastward, suggesting that the eastern extreme of the corridor has been less dynamic. Thus, the observed shift did not occur because cranes began using novel areas east of the centerline.

Reasons for these shifts throughout over half of the migratory corridor could be related to ecological phenomena or biases in data collection. Although opportunistic sightings have been identified as subject to errors based on variable precision of reported sightings [43], we believe precision differences were modest when compared to estimates we derived and had little influence on our results. Opportunistic sightings also have potential bias because of haphazard sampling [15,26]. When comparing opportunistic sightings and telemetry data gathered over the same years, median locations for sightings were consistently east of telemetry locations in all analysis windows at modest magnitudes of ≤15km and, in all but one instance, differences were not determined to be different from 0. Moreover, our analysis method provided a means to assess potential bias in opportunistic sightings. We derived prior distributions of slope parameters from initial analyses of opportunistic sightings exclusively, and updated those priors with information from telemetry data. Prior and posterior distribution means changed magnitude direction only twice and both had 95% credible intervals that included 0 (S1 Table). Therefore, general conclusions that could be drawn from opportunistic sighting data only were generally confirmed when considering telemetry data. As example, this was the case for analysis window 6, in which we found a median difference of 9.6 km between opportunistic sighting and telemetry data, yet posterior distributions for each analysis differed little from priors (S1 Table).

From an ecological perspective, changes in the migration corridor were likely related to how birds reacted to changes in key resources. Migration routes for midcontinent lesser snow geese (*Chen caerulescens caerulescens*) have shifted from a pathway along the Missouri River to one that includes a stopover in the Rainwater Basin Area in southcentral Nebraska where abundant waste corn and roost wetlands exist [44,45]. For whooping cranes, we speculate that the migration route changed because of modified patterns in surface water availability or upland habitat composition (e.g., crop type), which serve as primary stopover habitat for whooping cranes [19]. If surface water availability decreased in the western part of the migration corridor in response to changes in precipitation patterns or other factors [46], whooping cranes may have shifted areas used eastward in search of this critical resource, with cultural and social learning reinforcing this shift.

Similar to reasons for the route shift, any potential consequences of corridor change are speculative. Continuation of an easterly shift in the migration corridor may result in whooping cranes traveling through areas with less public knowledge and education of this endangered species, with fewer protected lands, and using a route that will increase total travel distance between wintering and breeding sites. Eastern parts of the Dakotas and Nebraska have been modified to a greater extent by human activities than western portions of these states, with higher rates of grassland conversion to cropland and wetland drainage [47,48]. Given these landscape modifications, whooping cranes may find fewer suitable stopover sites east of the current migration corridor, making further eastward shifts less likely. Continued reduced use of the western side of the corridor may suggest reduced availability of suitable stopover habitat, which would be a cause for concern if population growth declined from its current rate. However, the corridor shift demonstrates individual variation and behavioral flexibility, which would seem to be a desirable characteristic for a wetland-dependent species migrating through the Great Plains where periodic droughts occur and may occur with greater future frequency and severity [49]. Continued plasticity in migration behaviors and routes could maintain resistance of the population and species given changing landscape conditions. Considering current positive population growth rate [50], there is little evidence that the easterly shift and modest contraction are cause for immediate concern to species recovery, yet long term consequences are unknown.

Of greater certainty is that conservation organizations may need to consider refocusing efforts based on this updated corridor. Government agencies and non-governmental organizations use the migration corridor to target habitat projects, protection activities, and education. Agencies may want to increase efforts in landscapes previously not prioritized while decreasing efforts in areas that historically received greater focus. Shifting species ranges makes targeted conservation and mitigation difficult, because threats outside the range today could occur within it in the future [35]. Use for conservation planning and management of the largest corridor we identified that included all migration locations since the 1940s would provide a hedge against modest future shifts and interannual variability in habitat conditions for this endangered species.

## Supporting information

S1 Fig Migration corridors for whooping cranes of the Aransas-Wood Buffalo population, delineating 95% core migration areas. Corridors were initially delineated using opportunistic sighting data only collected from 1942–2016 (A) and telemetry data collected 2010–2016 (B). A combined corridor was calculated as a weighted average of the two (C).
(TIF)

S1 Table Results of analyses to determine changes in characteristics of the whooping crane migration corridor, 1942–2016. (DOCX)
