# Supplementary material for: Delineating and identifying long-term changes in the whooping crane (Grus americana) migration corridor
Source: PLoS One. 2018 Feb 15;13(2):e0192737. doi: 10.1371/journal.pone.0192737 (PMC5813961; doi:10.1371/journal.pone.0192737)
Supplement: S1 Table — (DOCX) [file pone.0192737.s002.docx]

S1 Table. Results of analyses to determine changes in characteristics of the whooping crane migration corridor, 1942–2016.

|  |  | Prior^a^ | | |  | Posterior^b^ | | |
| --- | --- | --- | --- | --- | --- | --- | --- | --- |
| Analysis window^c^ | Analysis^d^ | Estimate^e^ | LCI^f^ | UCI^g^ |  | Estimate | LCI | UCI |
| 1 | Directional Shift | 1.24 | -1.27 | 3.75 |  | 0.81 | -0.77 | 2.38 |
| 2 | Directional Shift | 0.18 | -2.02 | 2.39 |  | 0.21 | -1.14 | 1.56 |
| 3 | Directional Shift | 0.46 | -1.39 | 2.32 |  | 0.81 | -0.34 | 1.94 |
| 4 | Directional Shift | 1.05 | 0.41 | 1.68 |  | 1.05 | 0.65 | 1.45 |
| 5 | Directional Shift | 0.44 | -0.06 | 0.92 |  | 0.49 | 0.17 | 0.81 |
| 6 | Directional Shift | 1.66 | 1.17 | 2.13 |  | 1.66 | 1.35 | 1.97 |
| 7 | Directional Shift | 2.88 | 2.14 | 3.61 |  | 2.84 | 2.37 | 3.31 |
| 8 | Directional Shift | 1.19 | 0.70 | 1.69 |  | 1.02 | 0.71 | 1.34 |
| 9 | Directional Shift | 1.35 | 0.83 | 1.86 |  | 1.24 | 0.92 | 1.57 |
| 10 | Directional Shift | 0.21 | -0.14 | 0.55 |  | 0.28 | 0.06 | 0.50 |
| 11 | Directional Shift | 0.49 | -0.25 | 1.25 |  | 0.34 | -0.12 | 0.81 |
| 12 | Directional Shift | 2.75 | -1.92 | 7.41 |  | 2.11 | -0.14 | 4.37 |
| 13 | Directional Shift | -1.90 | -12.51 | 8.99 |  | -2.18 | -6.29 | 1.95 |
|  |  |  |  |  |  |  |  |  |
| 1 | Total width | 1.56 | -0.37 | 3.49 |  | 0.93 | -0.28 | 2.13 |
| 2 | Total width | -0.42 | -2.12 | 1.28 |  | -0.57 | -1.59 | 0.45 |
| 3 | Total width | 0.71 | -0.70 | 2.15 |  | -0.16 | -1.04 | 0.70 |
| 4 | Total width | -0.80 | -1.29 | -0.31 |  | -0.71 | -1.01 | -0.40 |
| 5 | Total width | -0.52 | -0.90 | -0.15 |  | -0.33 | -0.57 | -0.08 |
| 6 | Total width | -0.62 | -1.00 | -0.26 |  | -0.55 | -0.79 | -0.31 |
| 7 | Total width | -1.32 | -1.89 | -0.76 |  | -1.29 | -1.65 | -0.93 |
| 8 | Total width | -0.57 | -0.95 | -0.19 |  | -0.52 | -0.76 | -0.28 |
| 9 | Total width | -0.44 | -0.83 | -0.04 |  | -0.45 | -0.69 | -0.20 |
| 10 | Total width | -0.80 | -1.06 | -0.54 |  | -0.76 | -0.93 | -0.59 |
| 11 | Total width | 0.22 | -0.35 | 0.80 |  | 0.06 | -0.29 | 0.42 |
| 12 | Total width | 3.85 | 0.26 | 7.44 |  | 1.75 | 0.08 | 3.42 |
| 13 | Total width | -1.91 | -10.07 | 6.47 |  | 0.03 | -2.92 | 3.00 |
|  |  |  |  |  |  |  |  |  |
| 1 | West width | -1.32 | -4.43 | 1.80 |  | -0.63 | -2.48 | 1.25 |
| 2 | West width | 0.10 | -2.62 | 2.80 |  | 0.35 | -1.27 | 1.95 |
| 3 | West width | -2.28 | -4.14 | -0.37 |  | -1.21 | -2.34 | -0.06 |
| 4 | West width | 1.43 | 0.56 | 2.31 |  | 1.41 | 0.89 | 1.94 |
| 5 | West width | 0.52 | -0.08 | 1.10 |  | 0.40 | 0.02 | 0.77 |
| 6 | West width | 1.00 | 0.48 | 1.52 |  | 1.03 | 0.71 | 1.36 |
| 7 | West width | 1.45 | 0.67 | 2.25 |  | 1.41 | 0.93 | 1.90 |
| 8 | West width | 1.06 | 0.49 | 1.64 |  | 0.87 | 0.52 | 1.23 |
| 9 | West width | 0.50 | -0.08 | 1.07 |  | 0.48 | 0.13 | 0.83 |
| 10 | West width | 0.94 | 0.52 | 1.36 |  | 0.97 | 0.70 | 1.23 |
| 11 | West width | -0.57 | -1.48 | 0.32 |  | -0.38 | -0.91 | 0.14 |
| 12 | West width | -3.15 | -7.88 | 1.58 |  | -1.12 | -3.29 | 1.04 |
| 13 | West width | NA^h^ | NA | NA |  | -0.73 | -0.85 | -0.61 |
|  |  |  |  |  |  |  |  |  |
| 1 | East width | 1.61 | -0.72 | 3.98 |  | 1.05 | -0.43 | 2.53 |
| 2 | East width | -0.70 | -2.65 | 1.30 |  | -0.80 | -2.01 | 0.42 |
| 3 | East width | -0.13 | -2.56 | 2.31 |  | -1.17 | -2.70 | 0.35 |
| 4 | East width | 0.11 | -0.44 | 0.66 |  | 0.26 | -0.09 | 0.61 |
| 5 | East width | -0.36 | -0.81 | 0.10 |  | -0.11 | -0.42 | 0.19 |
| 6 | East width | 0.55 | 0.01 | 1.09 |  | 0.67 | 0.31 | 1.01 |
| 7 | East width | -0.60 | -1.63 | 0.43 |  | -0.55 | -1.24 | 0.14 |
| 8 | East width | 0.12 | -0.36 | 0.59 |  | 0.05 | -0.25 | 0.35 |
| 9 | East width | -0.23 | -0.77 | 0.29 |  | -0.28 | -0.62 | 0.06 |
| 10 | East width | -0.64 | -0.94 | -0.33 |  | -0.54 | -0.74 | -0.34 |
| 11 | East width | -0.27 | -1.02 | 0.50 |  | -0.39 | -0.86 | 0.09 |
| 12 | East width | NA | NA | NA |  | -2.14 | -3.62 | -0.67 |
| 13 | East width | -1.96 | -8.94 | 5.15 |  | -1.26 | -4.50 | 2.02 |

^a^ Resulting parameter estimates and 95% credible intervals of posterior distributions from initial analysis using opportunistic sightings only, which were used as prior distributions for a subsequent analysis using telemetry data.

^b^ Resulting parameter estimates and 95% credible intervals of posterior distributions from final analyses.

^c^ Spatial analysis windows (see Figure 2B).

^d^ Distribution shift analysis looked for temporal patterns in x-coordinate of whooping crane locations. Total width analysis looked for temporal patterns in distance of locations from their median value. West width analysis looked for temporal patterns in distance of locations west of a common median. East width analysis looked for temporal patterns in distance of locations east of a common median.

^e^ Beta estimate or slope parameter (km/year)

^f^ Lower 95% credible interval

^g^ Upper 95% credible interval

^h^ NA = no data available in analysis window to complete initial analyses.
